# Synaptic Plasticity Shapes Brain Connectivity: Implications for Network Topology

**DOI:** 10.3390/ijms20246193

**Published:** 2019-12-08

**Authors:** Mario Stampanoni Bassi, Ennio Iezzi, Luana Gilio, Diego Centonze, Fabio Buttari

**Affiliations:** 1 Unit of Neurology & Neurorehabilitation, IRCCS Neuromed, 86077 Pozzilli, Italyennio.iezzi@neuromed.it (E.I.); gilio.luana@gmail.com (L.G.); fabio.buttari@gmail.com (F.B.); 2 Laboratory of Synaptic Immunopathology, Department of Systems Medicine, Tor Vergata University, 00173 Rome, Italy

**Keywords:** brain networks, connectivity, synaptic plasticity, Alzheimer’s disease (AD), schizophrenia, long-term potentiation (LTP), synaptic scaling, resting state functional MRI (rs-fMRI)

## Abstract

Studies of brain network connectivity improved understanding on brain changes and adaptation in response to different pathologies. Synaptic plasticity, the ability of neurons to modify their connections, is involved in brain network remodeling following different types of brain damage (e.g., vascular, neurodegenerative, inflammatory). Although synaptic plasticity mechanisms have been extensively elucidated, how neural plasticity can shape network organization is far from being completely understood. Similarities existing between synaptic plasticity and principles governing brain network organization could be helpful to define brain network properties and reorganization profiles after damage. In this review, we discuss how different forms of synaptic plasticity, including homeostatic and anti-homeostatic mechanisms, could be directly involved in generating specific brain network characteristics. We propose that long-term potentiation could represent the neurophysiological basis for the formation of highly connected nodes (hubs). Conversely, homeostatic plasticity may contribute to stabilize network activity preventing poor and excessive connectivity in the peripheral nodes. In addition, synaptic plasticity dysfunction may drive brain network disruption in neuropsychiatric conditions such as Alzheimer’s disease and schizophrenia. Optimal network architecture, characterized by efficient information processing and resilience, and reorganization after damage strictly depend on the balance between these forms of plasticity.

## 1. Introduction

The functional properties of the brain are largely determined by the characteristics of its neurons and the pattern of synaptic connections between them. In the last century, how information is coded within a neuron and flows between neurons through synapses has been deeply investigated. However, little is known about how neural plasticity shapes network organization.

Comprehension of brain networks organization has been fueled by the application of procedures able to investigate brain connectivity in vivo [1,2] based on new theoretical/mathematical approaches (i.e., graph theory) to extract several measures that describe network architecture and functioning [3,4]. This approach led to the identification of specific features of brain networks, such as modularity and the presence of network hubs, that provide efficient information processing and elevated resistance to damage [5]. Furthermore, studies in patients with neurological diseases offered the chance to explore brain network reorganization following different types of damage [6,7,8].

Synaptic plasticity refers to the ability of neurons to modify the strength of their connections and is an important neurophysiological process involved in brain networks development and reorganization after damage [9]. Plasticity and network organization are highly intermingled, although they are generally studied as independent phenomena. Different forms of synaptic plasticity, namely, anti-homeostatic (i.e., Hebbian) and homeostatic plasticity (i.e., synaptic scaling), have been described. A fine balance between these two forms of synaptic plasticity could be crucial to maintain an optimal brain network architecture [9]. 

In the present review article, we will try to link together evidence from different research fields on the relationship between synaptic plasticity and principles of brain network organization. We will suggest that some key features of brain networks architecture may result from the fine tuning between different forms of synaptic plasticity. This perspective may be helpful to understand how networks adapt in response to brain damage and to explain mechanisms of network disruption in neuropsychiatric conditions such as Alzheimer’s disease and schizophrenia.

## 2. Brain Network Organization

Classical studies explored neuronal connections using reconstructions of electron micrographs of serial sections to map neuronal local connectivity and have been used to reconstruct the structure and connectivity of simple nervous systems, such as that of *Caenorhabditis elegans* and *Drosophila* [4,10,11]. The application of new neurophysiological and imaging tools offered the opportunity to study the activity of different brain areas simultaneously, with different degrees of spatial and temporal resolution. Electroencephalography (EEG) and magnetoencephalography (MEG) can be used to analyze functional connectivity (FC) through the analysis of temporal correlations between spontaneous activity of different regions and are characterized by elevated temporal resolution. Functional MRI (fMRI) has a higher spatial resolution and represents the most used approach to explore brain network organization in vivo [12,13]. Resting state-fMRI (rs-fMRI) offers the chance to explore overall brain connectivity in healthy individuals and in different pathological conditions [14,15]. In fMRI studies, FC is calculated by the analysis of temporal correlations between spontaneous activity of blood-oxygen-level-dependent (BOLD) signals coming from different brain regions [2,16]. Conversely, structural connectivity (SC) can be calculated using fiber tractography from diffusion tensor imaging (DTI) or studying the correlations in cortical thickness between areas. Anatomically connected regions show stronger FC [17,18]; however, functional interactions may also occur in brain areas not directly connected [17,18,19], and therefore, the relationship between SC and FC requires further investigation.

Network-based studies use SC and FC data to create a comprehensive map of brain connections [20,21,22]. The graph theory represents the most useful approach to model brain networks [4,23]. According to the graph theory, a complex network can be represented as a set of nodes and edges, respectively indicating the basic elements of the network and the relationships between them. This approach can be used to describe complex networks with different spatial resolution, from microscale to large-scale networks [1,5]. In large-scale networks, nodes can represent EEG channels or regions of interest identified on MRI. The definition of edges originates from the analysis of SC and FC between nodes. The structure of a graph can be further analyzed to extract a set of quantitative measures describing specific properties of the network, including global and local efficiency, modularity, and degree distribution [5]. An important parameter is the wiring cost, expressing the energetic expenditure due to fiber tracts length and number of synaptic connections [4]. 

Brain networks typically show a small-world topology characterized by the prevalence of high locally connected nodes with a relatively small number of long-distance connections, optimizing efficient network communication and limiting wiring costs increase [24]. A simple measure able to provide essential information about brain network organization is node degree, that is the number of connections to a single node. The degree distribution of a graph P(k) can be defined as the fraction of nodes having degree k. Brain networks show scale-free degree distribution, with a large prevalence of nodes with low degree and a small number of highly connected nodes, termed hubs, that support efficient global information processing [25]. Furthermore, brain networks are organized into modules composed of locally connected nodes, with long-distance connections between modules mainly confined to hub regions [26]. Such organization supports both functional segregation of information, which is provided by local communities of neurons highly interconnected, and integration, which measures the efficiency of global information transfer and the ability of networks to integrate distributed information [27]. 

Hubs are critical to provide efficient integration. Moreover, hubs show high inter-connectivity generating a subnetwork of densely interconnected hub regions, the rich club, which critically contribute to network efficiency and resilience to damage [4,26]. According to the preferential attachment theory, during the development of brain networks, the formation of hubs mostly follows a “rich get richer” principle, that is, the more connected nodes have greater chances to further increase their connectivity [28].

Considering the energetic expense of hyperconnectivity, this network architecture represents an efficient tradeoff between cost and efficiency, concentrating most connections to selected strategic nodes [24]. Accordingly, in invertebrates and mammalians, the brain shows the same organizational principles. Similar characteristics have been identified in several real-world complex networks, and this architecture provides high resilience to random damage due to the numerical prevalence of low-connected nodes [5,29]. Conversely, targeted attack to hubs may dramatically impact overall network efficiency [30], as evidenced in specific neuropsychiatric conditions such as Alzheimer’s disease (AD) [31] or schizophrenia [32,33].

## 3. Synaptic Plasticity

Neurons can modify the efficacy of synaptic connections through different forms of synaptic plasticity, including anti-homeostatic and homeostatic mechanisms [9,34]. 

Long term potentiation (LTP) is one of the most studied forms of synaptic plasticity and has been associated to learning and memory processes [35,36], as well as to clinical recovery after brain damage [37]. LTP has been extensively investigated in hippocampal neurons [34,35] and consists of persisting enhancement of synaptic excitability, accompanied by structural rearrangement occurring at both the presynaptic and post-synaptic terminal [38,39]. LTP induction is associated to remodeling of dendritic spines, including increased spine volume, stability and clustering [40,41,42]. LTP depends on the activation of *n*-methyl-d-aspartate receptors (NMDARs) [43,44], and some key properties of this form of synaptic plasticity directly arise from the functional characteristics of this receptor [38,45]. LTP is cooperative, as the concomitant activation of multiple synapses favors the induction of this form of synaptic plasticity. Moreover, LTP is associative, meaning that a weak stimulus can be reinforced if associated with a strong one. Finally, LTP is input-specific, as only activated synapses in a neuron undergo potentiation. One important characteristic is that LTP is associated to increased neuronal excitability, which in turn facilitates further induction of LTP in a positive feedback loop, making LTP an anti-homeostatic phenomenon [9]. It is important to mention that activation of NMDARs could also induce a different form of anti-homeostatic plasticity, represented by long-term depression (LTD), which is associated to a lasting reduction in synaptic excitability [9,46]. Moreover, LTD induction is associated to changes in dendritic spine morphology, including marked spine shrinkage leading to the elimination of dendritic spines [47,48]. Notably, LTD-induced spine retraction could be reversed by subsequent LTP [48]. Although through opposite effects, LTP and LTD mutually interact to refine neural connections during the development and to regulate cognitive processes.

**Figure 1:**
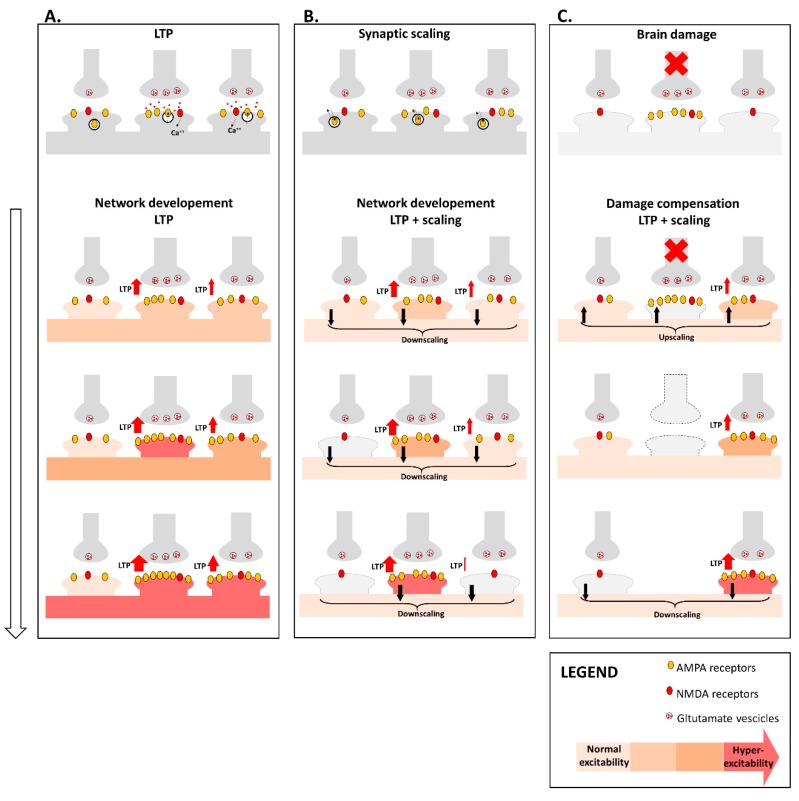
Different forms of synaptic plasticity cooperate to regulate neuronal activity. (**A**) LTP is input specific as it involves only active synapses. This form of NMDA-mediated plasticity implies calcium entrance in the post-synaptic terminal, which in turn induces amplified expression of AMPA receptors and increase of synaptic excitability, favoring further LTP expression. LTP is an anti-homeostatic form of plasticity and could promote uncontrolled enhancement of synaptic activity, leading to neuronal hyperexcitability and network instability during brain networks development. (**B**) Synaptic scaling is a homeostatic form of plasticity independent of NMDA receptors activation, involves all synapses and is mediated by increased (upscaling) or decreased (downscaling) expression of AMPA receptors. A balance between anti-homeostatic and homeostatic plasticity could promote optimal network organization associated to efficient information processing, with coexistence of potentiated and silent synapses (grey spines), allowing specific and segregated information processing, preventing excessive increase of overall excitability. (**C**) Brain damage induces acute disconnection depriving neurons of their synaptic inputs. Synaptic scaling and LTP may cooperate to restore neuronal excitability, promoting initial hyperexcitability (induced by compensatory upscaling) and favoring chronic reorganization properly balancing homeostatic and anti-homeostatic plasticity. Abbreviations: long-term potentiation (LTP); *N*-methyl-d-aspartate (NMDA); α-amino-3-hydroxy-5-methyl-4-isoxazolepropionic acid (AMPA).

Anti-homeostatic plasticity alone can lead to uncontrolled increases or decreases of neuronal excitability (Figure 1A). The total amount of excitatory drive toward a neuron must be tightly regulated, which is difficult to obtain if synapses are independently modified; therefore, other mechanisms are required to stabilize neuronal activity. Persistent increase or decrease of neuronal excitability is associated to compensatory synaptic scaling (Figure 1B). Synaptic scaling of excitatory synapses is not regulated by NMDARs and mainly relies on the activity of α-amino-3-hydroxy-5-methyl-4-isoxazolepropionic acid receptors (AMPARs). Changes in the expression and clustering of AMPARs induce an increase (upscaling) or decrease (downscaling) of neuronal excitability in response to opposite changes in the strength of their synaptic excitatory inputs [49,50]. Trafficking of surface AMPARs is regulated by the expression of Arc/Arg gene [51,52]. Unlike LTP, synaptic scaling is a homeostatic negative feedback mechanism and represents a form of hetero-synaptic plasticity, as it lacks input-specificity and involves all synapses of a given neuron. 

Homeostatic and anti-homeostatic plasticity may cooperate to maintain proper neuronal activity, preventing hyper- or hypoexcitability and concur to reestablish neuronal activity after brain damage (Figure 1C). LTP induction can be influenced by overall neuronal excitability so that synaptic upscaling or changes in inhibitory balance may increase synaptic activity favoring LTP expression [53,54]. Accordingly, after brain damage, reduced GABAergic transmission in surrounding neurons could promote functional reorganization [55,56]. Furthermore, downscaling of excitatory synapses is important to prevent chronic hyperexcitability and to promote, in concert with LTP, selective increases of synaptic activity.

## 4. Synaptic Plasticity and Brain Network Organization

Addressing the relationship between synaptic plasticity and brain network organization is particularly difficult due to multiple reciprocal influences between brain network structure and function. Indeed, network architecture strongly influences neuronal activity [57,58,59,60,61,62,63,64], and patterns of neuronal activity may differently shape synaptic connections. 

A fundamental characteristic of neuronal networks is the ability to produce rhythmic oscillations in different frequency ranges, providing integration of brain functioning in physiological conditions such as those occurring during sleep and awake [65,66]. In particular, temporal and spatial variations of frequency are useful to obtain coordinated information processing during sensory, motor and cognitive activities which are subserved by synchronous oscillations of neuronal networks [67,68,69]. The excitability state of neurons changes during oscillations so that firing probability is higher during the depolarizing phase, whereas during the hyperpolarizing phase, neurons show less propensity to fire in response to excitatory inputs [70,71]. Synchronous bursting of neuronal population may induce long-lasting changes in connectivity. In particular, high-frequency bursting induces LTP, whereas low-frequency activity is associated to LTD [35,72,73,74]. Furthermore, the temporal correlation between converging inputs to neurons can bidirectionally modulate synaptic strength according to the so-called spike timing-dependent plasticity (STDP). In particular, the firing of the presynaptic neuron can respectively induce LTP or LTD if occurring shortly before or after the firing of the postsynaptic neuron [75,76]. STDP provides an additional rule for Hebbian plasticity based on the temporal association of converging activity, bridging brain network organization and neuronal activity. This mechanism is required to form neuronal communities showing high connectivity and strongly coordinated activity during specific processing [77]. Oscillatory activity and STDP interact to shape effective coupling between anatomically connected areas. Intriguingly, the degree of synchrony of neuronal discharges and neuronal firing rate could be independently adjusted [78]. Accordingly, neurons cycling in phase with each other tend to show synchronized activity favoring synaptic LTP [79].

Previous synaptic history and state of neuronal excitability further complicate the relationship between neuronal activity and connectivity. It has been demonstrated that repeated synaptic activation may influence subsequent induction of Hebbian plasticity. In particular, considering that low and prolonged calcium entry is associated to LTD whereas high calcium influx likely mediates LTP [80], it has been proposed that changes in calcium levels into dendritic spines can modify plasticity induction [81]. 

Similarities existing between synaptic plasticity mechanisms and specific features of brain network organization suggest that different forms of plasticity could be directly involved in generating specific brain networks characteristics. LTP is anti-homeostatic, input-specific, activity-dependent and associative. Due to its properties, LTP could be directly implicated in generating highly connected nodes, allowing the establishment of strong and specific connections by independently acting at each synapse [82]. In particular, the preferential attachment theory of hub formation suggests the existence of an associative, positive feedback mechanisms which strongly follow the Hebbian plasticity rules. 

Experimental studies in rats have elegantly shown that LTP induction in the perforant pathway induced remodeling of hippocampal long-range connections [83,84]. In particular, increased interhemispheric communication and increased connectivity has been found between the hippocampus, the prefrontal cortex and the nucleus accumbens [83,84]. These data suggest that network effects of LTP induction at hubs’ level is associated to long-lasting widespread network remodeling of brain connectivity. 

It has been consistently shown that the isolated effect of anti-homeostatic positive feedback plasticity leads to perturbation in the stability of neuronal networks, that must be counterbalanced by negative feedback homeostatic plasticity to maintain network activity in an optimal range [9,85]. While LTP is anti-homeostatic and represents a possible substrate for hubs generation, we propose that homeostatic plasticity, in particular synaptic scaling, may intervene to maintain low connectivity (but still some connectivity) in the peripheral nodes of the network. 

The study of brain network reorganization in response to brain damage could help to shed light on the relationship between synaptic plasticity and brain network remodeling. Particular neuropsychiatric conditions, such as schizophrenia and AD, in which altered plasticity is one main neurophysiological feature [86,87], may represent useful models to investigate how synaptic plasticity alterations impact brain network architecture. Notably, the central role of plasticity alteration has also been proposed in other neurological conditions such as temporal lobe epilepsy. In epileptic models, the recurrence of seizures has been associated with imbalanced excitatory and inhibitory synaptic transmission leading to hypersynchronized neuronal activity [88,89], and epileptogenesis has been linked to altered expression of hippocampal LTP and LTD at glutamatergic and GABAergic synapses, respectively [90].

### 4.1. Synaptic Plasticity Promotes Brain Network Reorganization after Damage

Both brain network architecture and synaptic plasticity play an important role in clinical compensation of brain damage. As previously discussed, specific characteristics of brain networks organization provide high resistance to random damage [5]. Notably, anti-homeostatic and homeostatic plasticity could be both involved in promoting an efficient network reorganization after brain damage [91,92].

**Figure 2:**
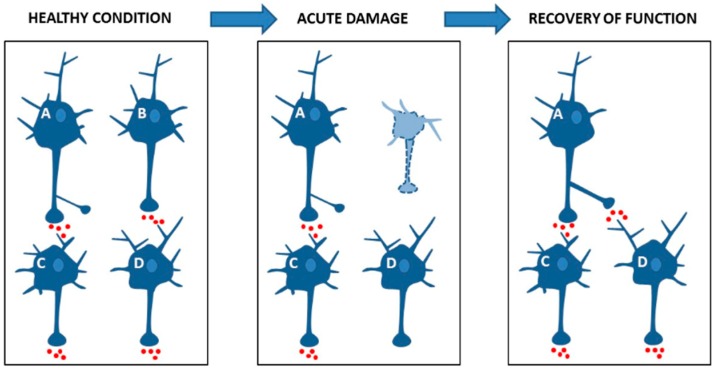
Synaptic plasticity promotes recovery after neural damage. Healthy condition: a schematic model representing neuronal excitatory connections. Neuron C and D receive synaptic excitatory inputs from neurons A and B respectively. Acute damage: damage to neuron B deprives neuron D of excitatory synaptic input leading to disconnection and symptoms appearance. Recovery of function: clinical recovery is associated to increased excitability of the surviving A neuron that unmasks latent synaptic connections through LTP and restores synaptic activity of neuron D.

Experimental studies pointed out that the efficiency of synaptic plasticity, and particularly of LTP, critically influences clinical recovery (Figure 2). In animal models of brain damage (i.e., focal ischemia), symptoms compensation relies on the ability of surviving neurons to increase their excitability, as shown by a positive correlation between improvement in clinical scores and increased excitatory glutamatergic transmission in perilesional area [37]. Synaptic plasticity can be explored non-invasively in humans using transcranial magnetic stimulation (TMS). It has been demonstrated that the amount of LTP-like plasticity inducible with different TMS protocols after brain damage, the so-called LTP reserve, correlated with the degree of clinical recovery [93,94]. These results strongly suggest that LTP, specifically enhancing synaptic efficacy, is a fundamental requisite for network remodeling after brain damage. 

Widespread increase in brain functional connectivity represents a common response to different types of damage, including traumatic brain injury [95,96,97], stroke [98], Parkinson’s disease [99,100] and mild cognitive impairment (MCI) [101,102]. This early-phase adaptation could be useful to counterbalance connectivity decline and restore network functionality, delaying symptoms onset. It has been recently proposed that selective remodeling of hub connectivity could represent an efficient mechanism to restore network activity containing the wiring cost. The central role played by LTP in clinical recovery agrees with the role of hubs as the preferential site for connectivity increases, according to the positive feedback nature of both phenomena. This pattern of reorganization requires normal functioning network hubs, and a prominent involvement of hubs has been accordingly evidenced in different neurological conditions [8].

Importantly, connectivity increases should be tightly regulated to preserve an optimal tradeoff between cost and efficiency [8]. Homeostatic plasticity cooperates with LTP to determine optimal brain network reorganization in both acute and chronic remodeling. In the early phases, synaptic upscaling could induce widespread hyperexcitability favoring network hyperconnectivity, with the aim of partially restoring network efficiency. Moreover, neuronal hyperexcitability favors LTP induction at hub level, further increasing hubs connectivity. Homeostatic plasticity changes may therefore regulate Hebbian plasticity expression. This could be particularly relevant also in the late phases of network reorganization when efficient downscaling is needed to limit excessive connectivity increase, preventing chronic diffuse hyperconnectivity and selectively shaping hub remodeling. 

Another line of evidence, strongly suggesting a strict relationship between LTP induction and hub remodeling, arises from the paradigm of cognitive reserve. Accordingly, higher levels of education, cognitive abilities, occupation and physical activity have been correlated with reduced functional impact of brain structural damage as demonstrated in healthy aging subjects and in neurological patients [103,104,105,106,107]. In preclinical studies, environmental enrichment with physical, cognitive, and social stimuli improved the performance in different behavioral tasks exploring memory and learning [108] and enhanced LTP induction [109,110,111,112,113].

In humans, cognitive reserve has been linked to increased connectivity of hub regions. In healthy elder subjects, higher cognitive reserve correlated with increased metabolism and functional connectivity of the anterior cingulate cortex [114]. Similarly, higher cognitive reserve has been associated with enhanced functional connectivity in the left frontal cortex and reduced cognitive impairment in MCI and AD patients [115,116]. It has been proposed that cognitive reserve may promote brain network resilience increasing hubs connectivity, thus enhancing the resistance of hubs to damage [117,118].

### 4.2. Synaptic Plasticity Dysfunction May Drive Brain Network Disruption

AD and schizophrenia could represent useful models to explore the relationship between LTP expression and hubs connectivity. In particular, in AD and schizophrenia, impaired plasticity [87,119] may be responsible for reduced hubs degree and centrality, and decreased rich club connectivity [7,31,120]. In particular, impaired synaptic plasticity alters the synchrony of both local and distributed neuronal oscillations and could promote brain network dysfunction [69,121,122].

AD is a neurodegenerative disease characterized by accumulation of amyloid-β (Aβ) and tau protein [123] associated with prominent cognitive decline [124]. In the hippocampus of AD patients synaptic alterations have been evidenced since the early phases of the disease [125,126]. In particular, it has been proposed that early synaptic plasticity impairment could represent a main cause of memory deficits in AD, even independently of neurodegeneration [86,119]. Studies in animal models of AD documented lacking hippocampal LTP induction [127,128,129], and pathological LTD enhancement [130]. Accordingly, it has been observed that elevated levels of soluble Aβ oligomers could reduce LTP and promote LTD expression in the hippocampus [131,132,133,134]. It has been suggested that also impaired homeostatic plasticity could contribute to the clinical manifestations and disease progression in AD. In particular, altered interaction between homeostatic and anti-homeostatic plasticity in AD could ultimately promote synaptic loss [135,136]. In line with experimental data, in early AD patients TMS studies have confirmed that LTP-like plasticity is abolished, and LTD-like plasticity induction is favored [86,119].

Reduced small world topology and altered connectivity, particularly in associative areas, have been reported in AD [7,15,137,138,139,140,141,142]. In particular, it has been shown a specific involvement of hub regions [7,30,142,143] with decreased centrality of the hippocampus and the default mode network [31]. In addition, impaired hubs connectivity has been correlated with worse cognitive performance and reduced CSF levels of Aβ 1–42 [31]. It has been evidenced that hubs show increased Aβ deposition in MCI, AD and even in older healthy subjects [120,141,144,145]. In fact, a growing body of evidence leads to the hypothesis that chronic hyperconnectivity and enhanced neuronal activity could expose hubs to Aβ deposition and neurodegeneration [8,143]. Altered synaptic plasticity expression could explain the peculiar hubs vulnerability described in AD. In particular, impaired LTP and favored LTD may specifically disrupt hubs connectivity leading to compensatory maladaptive upscaling in healthy neighboring neurons, resulting in chronic hyperexcitability [135].

Schizophrenia is a highly disabling psychiatric condition characterized by positive and negative symptoms, including hallucinations and delusions, with most of the patients showing a progressive clinical decline [146]. The pathogenesis of schizophrenia has been classically linked to neurotransmitters alteration [147], neurodevelopmental disorders [148] and disconnection [149].

Recently, disrupted synaptic plasticity has been proposed as a possible pathophysiological marker of schizophrenia [87,150,151]. Accordingly, reduced spine density has been described in the prefrontal and temporal cortices of schizophrenic patients [152,153,154,155], and altered expression and function of NMDARs and AMPARs have been reported [156,157,158,159,160]. NMDARs dysfunction seems to be particularly relevant, as NMDAR antagonists could produce symptoms which strongly resemble schizophrenia manifestations [161]. Impaired LTP and LTD-like plasticity has been consistently reported in patients with schizophrenia [150,151,162]. Using a TMS protocol useful to explore cortical connectivity and in particular spike timing-dependent plasticity [163,164], reduced LTP-like plasticity has been shown between posterior parietal and frontal cortices in schizophrenia compared to control subjects [87].

Previous studies exploring brain network organization in schizophrenia showed alterations of several properties [165,166,167,168,169,170]. In particular, reduced hub connectivity [171] and rich club organization have been reported in schizophrenic patients [172]. Decreased connectivity within the frontal cortex has been considered a pathophysiological hallmark and decreased centrality in cortical and subcortical frontal areas has been reported accordingly [166,173,174]. In line with the disconnection hypothesis, impaired hub connectivity and reduced rich club efficiency may alter overall brain connectivity in schizophrenia [149].

Changes in synaptic plasticity expression may explain the hubs loss seen in schizophrenia. It has been suggested that spike timing-dependent plasticity alterations may be responsible of progressive disconnection of prefrontal circuits [175]. Altered NMDA-mediated synaptic plasticity reduces temporal correlation between converging inputs to connected neurons and could lead to additional activity-dependent disconnection of prefrontal networks [175]. In fact, subverted spike timing-dependent plasticity could induce LTD instead of LTP in prefrontal networks, ultimately producing progressive disruption of anterior hubs and generating a persistent disconnection within the rich club.

## 5. Conclusions

Synaptic plasticity mechanisms and specific features of brain network share common principles that contribute to explain how neural plasticity influences brain network organization. Indeed, different forms of synaptic plasticity could be directly involved in generating specific brain networks’ characteristics. A cooperative, associative, input-specific and anti-homeostatic Hebbian plasticity is well suited to form brain networks characterized by modules and hubs, providing segregation and integration of information. LTP could be implicated in generating highly connected nodes that are crucially involved in network remodeling after brain damage and also represent the specific target of pathophysiological processes in different neuropsychiatric conditions. Conversely, homeostatic forms of synaptic plasticity intervene to prevent excessive connectivity in the peripheral nodes, stabilizing network activity and preventing excessive cost-efficiency increase. Finally, the fine tuning between homeostatic and anti-homeostatic plasticity plays a key role in recovery after damage and may help to understand how brain networks reorganize in response to different neurological conditions. Further studies combining neurophysiological investigations and fMRI measures are required to better define the relationship between synaptic plasticity and brain network topology.

## Abbreviations

EEG	Electroencephalography
MEG	Magnetoencephalography
FC	Functional Connectivity
fMRI	Functional MRI
rs-fMRI	Resting state-fMRI
BOLD	Blood-Oxygen-Level-Dependent
SC	Structural Connectivity
DTI	Diffusion Tensor Imaging
P(k)	Degree Distribution of a Graph
AD	Alzheimer’s disease
LTP	Long term potentiation
NMDARs	N-methyl-D-aspartate receptors
LTD	Long-Term Depression
AMPARs	α-amino-3-hydroxy-5-methyl-4-isoxazolepropionic acid receptors
STDP	Spike Timing-Dependent Plasticity
TMS	Transcranial Magnetic Stimulation
MCI	Mild Cognitive Impairment
Aβ	Amyloid-β
